# Endovascular Repair for Giant Right Subclavian Artery Aneurysm With Gore Viabahn and Pull-Through Procedure: A Case Report

**DOI:** 10.3389/fsurg.2022.939818

**Published:** 2022-07-05

**Authors:** Binshan Zha, Zhiyong Chen, Huan Ou-yang

**Affiliations:** Department of Vascular and Thyroid Surgery, Department of General Surgery, The First Affiliated Hospital of Anhui Medical University, Hefei, China

**Keywords:** aneurysm, subclavian artery, endovascular repair, case report, stent graft

## Abstract

**Background:**

Giant true subclavian artery aneurysms (SAAs) (>5 cm) are rare. Technical and anatomical considerations complicate the endovascular treatment of SAAs and pose some challenges. Here, we present a giant right SAA that was successfully excluded using stent grafts with the pull-through technique after two interventional steps and discuss the pull-through technique details as well as the lessons to be learned from this case.

**Methods:**

A 50-year-old man presented at our department complaining of dyspnea and hoarseness. Computed tomography angiography (CTA) showed a giant right SAA with partial intraluminal thrombus and severe angulated aneurysm necks originating from the proximal right subclavian artery, approximately 70 × 71 mm in size.

**Outcomes:**

An 8 × 100-mm Gore Viabahn was selected to exclude the SAA. A decision was made to stabilize the wire tension using the pull-through technique. Final angiography showed that the SAA was essentially excluded, and slight endoleak was observed. At 6 months, imaging showed that the aneurysm was not obviously shrinking, there was still an endoleak and stent graft dislodgement was observed. Angiography confirmed a type Ia endoleak, which was managed by the placement of a 10 × 50-mm Gore Viabahn, again with the assistance of the pull-through technique. At the 25-month follow-up, CTA showed that the SAA was satisfactorily excluded, with no endoleak, and the SAA was reduced in size.

**Conclusions:**

Endovascular treatment of SAAs is a safe, reliable and minimally invasive approach. The pull-through technique may improve wire tension and device stabilization. Additionally, size selection and positioning should be reappraised under a severely angulated aneurysm neck.

## Introduction

Subclavian artery aneurysms (SAAs) are rare peripheral aneurysms accounting for 1% of all aneurysms (1). Giant true SAAs (>5 cm) are rarer. Most cases are treated by open surgery (2, 3). With advances in interventional techniques, endovascular repair has been suggested as a less invasive alternative to surgery (4–10). However, technical and anatomical considerations complicate the treatment of SAAs and pose some challenges. Few case reports of giant SAAs that have been managed by endovascular treatment are described in the literature (7). Furthermore, studies on endovascular repair of SAAs with severely angulated aneurysm necks are lacking. Here, we discuss the details of the pull-through technique used to manage a giant SAA with angulated aneurysm necks and the lessons to be learned from this case.

## Case Presentation

**Figure 1:**
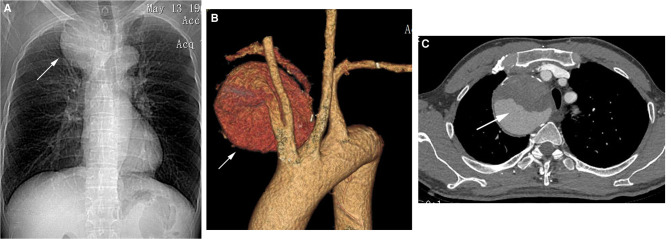
(**A**) Chest X-ray showed an intrathoracic mass in the upper lobe of the right lung and displacement of the trachea to the left (white arrow). (**B,C**) Computed tomography angiography (CTA) revealed a giant right subclavian artery aneurysm (SAA) with partial intraluminal thrombus and severe angulated aneurysm necks (white arrow).

A 50-year-old male presented at our department complaining of dyspnea and gradually worsening hoarseness of the voice over one month. There was no neurovascular compression and no sign of thoracic outlet syndrome. On admission, his erythrocyte sedimentation rate, C-reactive protein level, and other laboratory findings were normal. A chest X-ray showed an intrathoracic mass in the upper lobe of the right lung and displacement of the trachea to the left (Figure 1A).

Computed tomography angiography (CTA) revealed a giant right subclavian artery aneurysm (SAA) with partial intraluminal thrombus and severe angulated aneurysm necks originating from the proximal right subclavian artery, approximately 70 × 71 mm in size (Figures 1B, C). The proximal neck diameter of the aneurysm was 7 mm, and its distal neck diameter was 6 mm. The lengths of the proximal and distal landing zones of the aneurysm were sufficient (>30 mm). The right vertebral artery was tenuous, and the left vertebral artery was dominant. In light of the operative risk, based on the patient's choice and the wide application of endovascular repair, we proceeded with endovascular repair.

**Figure 2:**
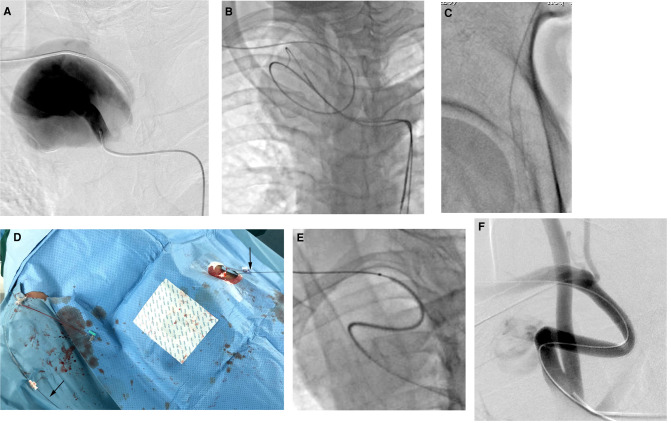
(**A**) Selective angiography *via* the left femoral artery verified the CTA findings. (**B**) The guidewire was circled along the shape of the aneurysm instead of along the shortest path. (**C**) The tip of the guide wire was introduced into a sheath placed in the femoral artery. (**D**) The guidewire was held out of the body, thus creating a through-and-through guide wire (black arrows). (**E**) The stent graft smoothly advanced to the intended position, 20 mm away from the orifice of the right subclavian artery. (**F**) Final angiography showed that the SAA was essentially excluded, although a slight endoleak was also observed.

Selective angiography *via* the left femoral artery verified the CTA findings. The distal subclavian artery was located at the lower back of the aneurysm, and the proximal and distal aneurysm necks presented with a type Z distortion (Figure 2A). A 6-Fr sheath was introduced into the right brachial artery. A 5F VER catheter with a 0.035-inch guidewire (Terumo, Tokyo, Japan) was advanced into the innominate artery. Because the aneurysm cavity was large, and the neck of the aneurysm was angulated, the guide wire was circled along the shape of the aneurysm instead of along the shortest path; after it was straightened, it bounced back (Figure 2B).

The pull-through technique was used to stabilize the wire tension and the device during the procedure (11). First, the guidewire was exchanged for a 0.035-inch stiff type guidewire (Terumo, Tokyo, Japan). Then, the tip of the guide wire was introduced into a 7-Fr introducer sheath placed in the femoral artery (Figure 2C). The guide wire was held outside of the body, thus creating a through-and-through guide wire (Figure 2D). We did not use a Lunderquist stiff wire (Cook Medical) to correct the distortion to reduce the risk of aneurysm rupture. Meanwhile, we measured the true and effective length of the SAA, using an intraoperative pigtail marking catheter, to be 70 mm. Because a combination of 10 × 50-mm and 8 × 50-mm Viabahn stent grafts (W. L. Gore & Associates, Inc, Flagstaff, Ariz) could have led to instability and an inadequate distal landing zone, and a discrepancy was present in the diameter of the proximal and distal landing zones, resulting in a 10 × 100-mm Viabahn stent graft being too oversized (60%) for the distal landing zone, we chose an 8 × 100-mm Viabahn stent graft as a compromise. Thus, an 8 × 100-mmViabahn stent graft (15% oversized) was delivered through the right brachial arteriotomy site after changing the initial 6-Fr sheath to an 8-Fr sheath. Two doctors held the ends of the guidewire in the two access sheaths and maintained the tension, and the third doctor advanced the stent graft smoothly without obvious resistance to the intended position, 2 cm away from the orifice of the right subclavian artery (Figure 2E). Final angiography showed that the SAA was essentially excluded and that the ﬂow of the right vertebral artery was preserved, and slight endoleak was observed, which appeared to arise from the inflexion of the stent graft (Figure 2F). Considering that some slight endoleaks have been observed to seal spontaneously, no further action was taken.

**Figure 3:**
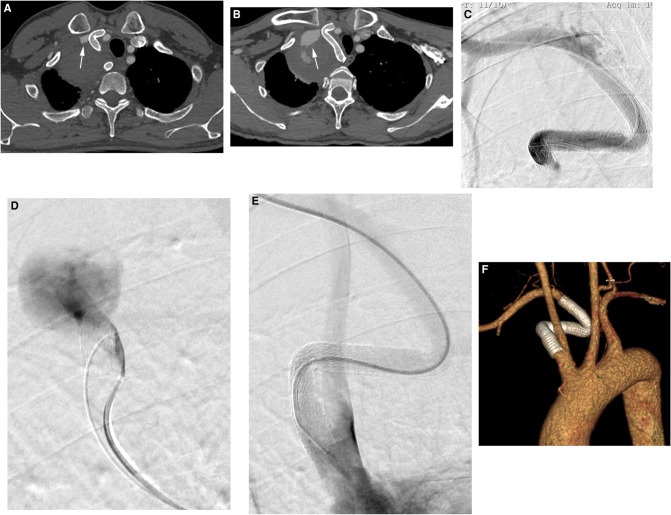
(**A**) CTA after 1 week indicated that the SAA was essentially occluded with a patent stent graft, and thrombus formation was observed in the aneurysm (white arrow). (**B**) At the 6-month follow-up, the aneurysm was not obviously shrinking, and CTA revealed that there was still an endoleak into the aneurysmal sac (white arrow). (**C,D**) Angiography revealed no contrast material leakage through the fabric of the inflexion of the stent graft, confirming a type Ia endoleak and the stent graft was caudally displaced. (**E**) Angiography showed successful exclusion of the aneurysm, with the absence of a type I endoleak. (**F**) At the 25-month follow-up, CTA revealed no evidence of an endoleak into the aneurysmal sac, and the stent graft was patent.

**Figure 4:**
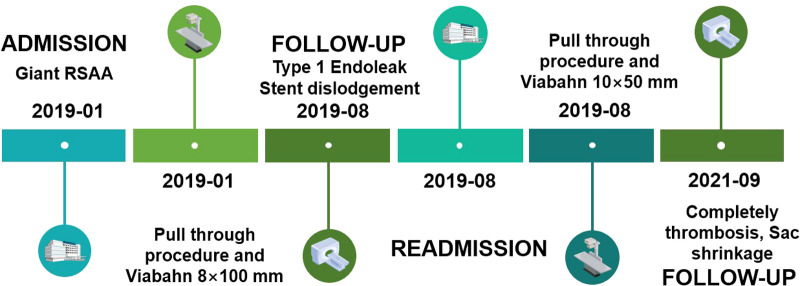
A timeline with relevant information from this case. RAAA: right SAA.

There were no procedure-related complications. CTA after 1 week indicated that the SAA was essentially occluded by the patent stent graft, and thrombus formation was observed in the aneurysm (Figure 3A). However, the endoleak did not disappear as expected, and imaging was continued due to low flow. At the 6-month follow-up, the aneurysm was not obviously shrinking, and CTA revealed that there was still an endoleak into the aneurysmal sac (Figure 3B). The patient was readmitted. We positioned a 5F VER catheter below the proximal fixation site in the main body of the stent graft. Angiography revealed no contrast material leakage through the fabric of the inflexion of the stent graft, confirming a type Ia endoleak (Figures 3C, D) and the stent graft was caudally displaced. The Ia endoleak was managed by the placement of a 10 × 50-mm Gore Viabahn (25% oversized) delivered through the left femoral puncture site (8-Fr sheath), again with the assistance of the pull-through technique. The proximal part of the stent graft was positioned at the orifice of the subclavian artery to extend the landing zone. Angiography showed the successful exclusion of the aneurysm, with the absence of a type I endoleak (Figure 3E). At the 25-month follow-up, CTA revealed no evidence of endoleak, complete thrombosis and that the SAA had decreased to 66 × 60 mm (Figure 3F). A timeline with relevant information from this case is shown in Figure 4.

## Discussion

Although open surgery remains effective for complex vascular diseases, advanced endovascular therapy has been preferred by vascular surgeons. Endovascular SAA repair is a technically feasible technique, useful in both elective and emergency cases. However, technical and anatomical considerations that complicate the endovascular treatment pose some challenges. Endovascular repair of SAAs is case-dependent. In some complex cases, the neck of the aneurysm is close to the proximal end of the subclavian artery, and a stent graft can be implanted using the simultaneous kissing stent technique to maintain the blood flow of the right subclavian and carotid arteries and to prevent a type 1 endoleak (10). Furthermore, in cases where the SAA is located in the arterial branches, a combination of stent graft and coil embolization has been applied for endovascular treatment (5). Furthermore, transcatheter closure has been suggested in selected patients using an Amplatzer vessel plug (7, 8). However, studies on endovascular repair of SAAs with severely angulated aneurysm necks are lacking. The support from the guidewire and the flexibility of the stent graft need to be considered, especially the latter in such cases. There are two methods available to improve guidewire support during the procedure: (1) utilizing an extra-stiff guidewire and (2) the buddy-wire technique (12). However, the feasibility of the procedure could be limited by lesion anatomy, such as tortuosity, which may increase the risk of intimal injury and aneurysm rupture. In addition, these approaches do not provide sustained and steady support force. Therefore, the pull-through technique was used, as this approach can provide steady and accurate support force.

In this case, the aneurysm presented with severely angulated proximal and distal aneurysm necks (type Z distortion). In addition, due to the limited space, the mobility of the right SAA was poor, and its Z distortion could not be corrected, even if the delivery system could be established. Stabilizing the wire tension was the crucial step. First, we created a through-and-through guidewire and used a Terumo stiff-type guidewire instead of an extra-stiff guidewire. Second, the three doctors must coordinate their activities to maintain accurate and constant tension control. In essence, the core of this technique is maintaining the tension of the guidewire. Third, stent selection is also an important issue. In this case, a flexible stent graft was a better option because it was easier to navigate the delivery of this type of device *via* the angulations in the aneurysm necks. To my knowledge, the Viabahn stent is a flexible covered stent that has a low profile and a highly flexible delivery system. It is also the preferred internal iliac artery covered stent for the modified sandwich-graft technique in complex cases in which it is used to preserve hypogastric flow during endovascular aneurysm repair. Therefore, in this case, the Viabahn stent was our preferred choice (13, 14).

There are several lessons to be learned from this case. The size selection and positioning of the stent graft should be given attention. Typically, 15%–20% oversizing is recommended for a stent graft for aneurysm, and the minimal length of the proximal landing zone is 15 mm. However, more oversized and extended proximal landing zones need to be taken into consideration when an angulated aneurysm neck or thrombosed aneurysm neck is involved. In this case, we selected a stent that was 8 mm in diameter and positioned it 2 cm away from the orifice of the right subclavian artery in the first operation, which resulted in a type I endoleak. During follow-up, this leak was erroneously assumed to be a slight type III endoleak because it seemed to originate from the inflexion of the stent graft. Furthermore, stent graft dislodgement was also observed. Eventually, it was resolved by introducing a larger stent graft with an extended landing zone.

Additionally, there are some details to note. It is suggested to perform the approach using a long introducer sheath to protect the Viabahn stent graft, especially when the Viabahn stent is deployed in the branch arteries during a fenestration procedure. Furthermore, it is generally recommended to advance the catheter from the second through-and-through access to protect the angulation zones while pulling the guidewire. In this case, our consideration during the procedure was that the stent advancement path was shorter from the brachial artery approach, and the stent graft should be advanced smoothly without obvious resistance. In addition, the kissing stent technique did not used which may sometimes cause the suture string of the Viabahn stent to become lodged and prevent deployment. Therefore, we did not use the above protective long introducer sheath and catheter. Fortunately, we managed to advance the catheter to the intended position. However, it is safer to use a protective long sheath and catheter while pulling the guidewire.

## Conclusion

We successfully treated a giant right SAA *via* the pull-through procedure and Gore Viabahn stent grafts, suggesting that this approach may provide a less invasive alternative to open surgery that improves wire tension and device stabilization. Additionally, size selection and positioning should be reappraised in cases of severely angulated aneurysm necks.

## Data Availability

The raw data supporting the conclusions of this article will be made available by the authors, without undue reservation.
